# Alzheimer's disease-related amyloid-*β* induces synaptotoxicity in human iPS cell-derived neurons

**DOI:** 10.1038/cddis.2015.72

**Published:** 2015-04-02

**Authors:** K Nieweg, A Andreyeva, B van Stegen, G Tanriöver, K Gottmann

**Affiliations:** 1 Institute of Neuro- and Sensory Physiology, Medical Faculty, Heinrich-Heine-University Düsseldorf, Düsseldorf, Germany; 2 Institute of Pharmacology and Clinical Pharmacy, Phillips University, Marburg, Germany

## Abstract

Human induced pluripotent stem cell (iPSC)-derived neurons have been proposed to be a highly valuable cellular model for studying the pathomechanisms of Alzheimer's disease (AD). Studies employing patient-specific human iPSCs as models of familial and sporadic forms of AD described elevated levels of AD-related amyloid-*β* (A*β*). However, none of the present AD iPSC studies could recapitulate the synaptotoxic actions of A*β*, which are crucial early events in a cascade that eventually leads to vast brain degeneration. Here we established highly reproducible, human iPSC-derived cortical cultures as a cellular model to study the synaptotoxic effects of A*β*. We developed a highly efficient immunopurification procedure yielding immature neurons that express markers of deep layer cortical pyramidal neurons and GABAergic interneurons. Upon long-term cultivation, purified cells differentiated into mature neurons exhibiting the generation of action potentials and excitatory glutamatergic and inhibitory GABAergic synapses. Most interestingly, these iPSC-derived human neurons were strongly susceptible to the synaptotoxic actions of A*β*. Application of A*β* for 8 days led to a reduction in the overall FM4–64 and vGlut1 staining of vesicles in neurites, indicating a loss of vesicle clusters. A selective analysis of presynaptic vesicle clusters on dendrites did not reveal a significant change, thus suggesting that A*β* impaired axonal vesicle clusters. In addition, electrophysiological patch-clamp recordings of AMPA receptor-mediated miniature EPSCs revealed an A*β*-induced reduction in amplitudes, indicating an impairment of postsynaptic AMPA receptors. A loss of postsynaptic AMPA receptor clusters was confirmed by immunocytochemical stainings for GluA1. Incubation with A*β* for 8 days did not result in a significant loss of neurites or cell death. In summary, we describe a highly reproducible cellular AD model based on human iPSC-derived cortical neurons that enables the mechanistic analysis of A*β*-induced synaptic pathomechanisms and the development of novel therapeutic approaches.

In Alzheimer's disease (AD), synapse damage and synapse loss are thought to underlie cognitive deficits.^1^ Oligomers of the amyloid-*β* (A*β*) peptide appear to induce synaptic failure as an early event in the etiology of AD.^2, 3, 4^ However, despite its well-established synapse-impairing effects in rodent models,^5, 6, 7^ the synaptotoxic actions of A*β* most relevant for the human disease have not been identified in a human model system. Several studies have investigated the synaptotoxic effects of A*β* in cultured rodent neurons and in transgenic mouse models revealing a multitude of potential mechanisms affecting synapses. Postsynaptic A*β* actions result in the loss of functional (*α*-amino-3-hydroxy-5-methyl-4-isoxazolepropionic acid (AMPA)-type) glutamate receptors,^8, 9, 10^ involve long-term depression-like mechanisms,^9, 11, 12^ and lead to the degradation of the entire postsynapse (dendritic spines).^9, 11, 13^ In addition, several distinct presynaptic A*β* actions on the synaptic vesicle cycle have been described.^10, 14^ Furthermore, A*β*-induced impairments of axonal transport regulation and A*β*-induced axon degeneration have been found in rodent neurons.^15, 16, 17^ This puzzling diversity of A*β*-induced synapse-related defects raises the question whether all of them are involved in the early pathomechanisms of human AD.

In addition to well-established animal systems, the modelling of human neurological disease pathologies by human induced pluripotent stem cell (hiPSC) technology^18^ has been proposed as an innovative approach.^19, 20, 21^ The *in vitro* differentiation of hiPSCs to excitable neurons has been reported using a variety of protocols.^22, 23, 24^ However, quantitative analysis of both functional glutamatergic and GABAergic synapses has been difficult to achieve.^19, 25, 26^ In addition to studying the functional properties of iPSC-derived human neurons from healthy individuals, the *in vitro* differentiation of patient-derived iPSCs has been used to model complex neurodevelopmental and neurodegenerative diseases.^19, 27, 28^ Recently, iPSCs derived from AD patients have been reported to exhibit increased secretion of A*β* upon *in vitro* neuronal differentiation; however, neither a loss of synapses nor an impairment of synapse function was detected.^21, 29, 30, 31, 32, 33^ Here we describe a hiPSC-based, carefully optimized *in vitro* differentiation protocol, including a novel immunopanning step, which enabled us to study the deleterious effects of application of A*β* on human cortical neurons and on human synapses.

## Results

### Neural differentiation of hiPSCs and immunopurification of hiPSC-derived immature neurons

**Figure 1:**
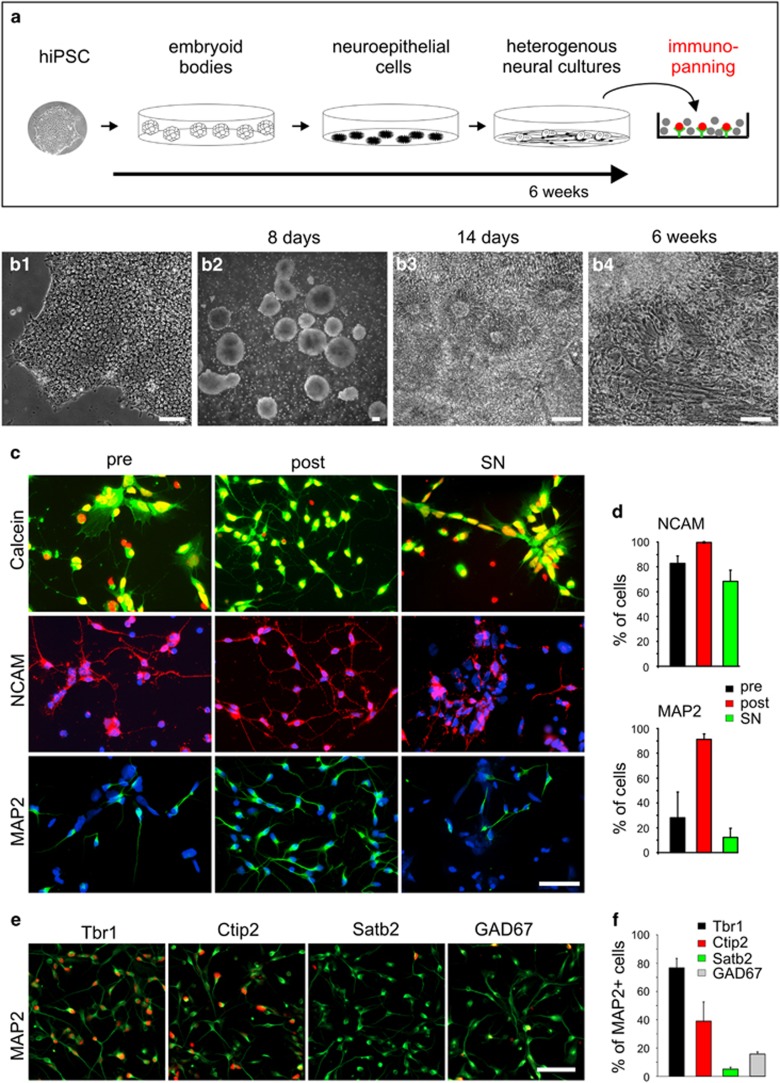
Purification of human iPSC-derived immature cortical neurons by immunopanning. (**a**) Scheme of *in vitro* differentiation of human iPSCs to neural cells prior to immunopanning. (**b**) Photomicrographs of distinct stages of *in vitro* neural differentiation of human iPSCs. **b1**, human iPSCs; **b2**, EBs; **b3**, neuroepithelial rosettes; **b4**, heterogeneous neural culture prior to immunopanning. Scale bars=100 *μ*m. (**c**) Efficiency of immunopanning. Cells (pre: heterogeneous dissociated culture without immunopanning; post: immature neurons isolated by immunopanning (with NCAM antibody, adherent cells); SN: cells present in supernatant after immunopanning (non-adherent cells)) were further cultured for 1 day after immunopanning. Top panel: Calcein (green) and DAPI (4,6-diamidino-2-phenylindole; red nuclei) co-stainings of hiPSC-derived cells. Middle/bottom panels: Co-immunostainings (with DAPI, blue nuclei) for NCAM (middle, red) and for MAP2 (bottom, green), respectively. Scale bar=50 *μ*m. (**d**) Quantification of NCAM (top) and MAP2 (bottom) immuno-positive cells (% of calcein stained cells), respectively, in dissociated cultures without immunopanning (pre), antibody adherent cells after immunopanning (post), and non-adherent cells after immunopanning (supernatant, SN). Ten minutes immunopanning with NCAM antibodies drastically enriched MAP2 immuno-positive immature neurons to >90% purity. (**e**) Co-immunostainings of purified immature neurons for MAP2 (green) to stain all neurons and for several cortical markers (red: Tbr1; Ctip2; Satb2) or GABAergic interneurons (red: GAD67). Cells were cultured for 1 day after immunopanning. Scale bar=50 *μ*m. (**f**) Quantification of immature neurons expressing the marker proteins indicated (percentage of MAP2 immuno-positive cells; three independent immunopannings). The majority of MAP2 immuno-positive neurons represented immature deep layer cortical neurons and GABAergic interneurons

hiPSCs were cultured (Supplementary Figure S1) and *in vitro* differentiated using an embryoid body (EB) system similar to published protocols.^22^ After initial differentiation, EBs were plated on a matrigel substrate leading to the formation of paired box protein 6 (Pax6)-expressing neuroepithelial rosettes (Supplementary Figure S2) that further differentiated to heterogeneous cultures also containing non-neuronal cells (Figures 1a and b). After 6–8 weeks of *in vitro* differentiation, heterogeneous cultures were dissociated to single cells, which were subjected to immunopurification. Classical immunopanning^34^ with specific modifications was performed using the neural cell adhesion molecule (NCAM) antibody VIN-IS-53 to isolate immature neurons expressing NCAM at a high level. To quantify immunopanning efficiency, dissociated cells without immunopanning (control), dissociated cells isolated by NCAM immunopanning, and dissociated cells non-adherent to the panning plates, respectively (Figure 1c), were immunocytochemically stained for NCAM and the neuronal marker microtubule-associated protein 2 (MAP2) 1 day after immunopurification (Figures 1d and e). The fraction of MAP2-positive cells was strongly increased in cells isolated by NCAM immunopanning (91.2±4.3%) as compared with control cells (28.1±20.6%) and to cells non-adherent to the panning plates (12.2±7.4%) (Figure 1g). The fraction of NCAM-positive cells was also increased by immunopanning (Figure 1f); however, as expected from the low level NCAM expression in neural precursor cells, the increase was less pronounced as compared with MAP2. We next characterized the immunopurified immature neurons using immunocytochemistry. Staining for cortical marker proteins revealed that the vast majority of MAP2-positive cells expressed markers of deep layer cortical neurons (Ctip2 (chicken ovalbumin upstream promoter transcription factor-interacting protein 2), Tbr1 (T-box, brain, 1)), while only 5.0±1.4% of the MAP2-positive neurons expressed the upper layer marker special AT-rich sequence-binding protein 2 (Satb2; Figures 1h and i). Similar to the composition of neuronal cell types in the *in vivo* cortex, 15.7±1.7% of the MAP2-positive neurons were GABAergic (glutamic acid decarboxylase 67 (GAD67) positive) (Figures 1h and i). Survival of immature neurons was not affected by the immunopanning procedure (Figure 1c). In summary, NCAM immunopanning of hiPSC-derived heterogeneous cultures resulted in highly purified MAP2-positive immature deep-layer cortical neurons.

### Morphological maturation and action potential generation in hiPSC-derived neurons purified by immunopanning

**Figure 2:**
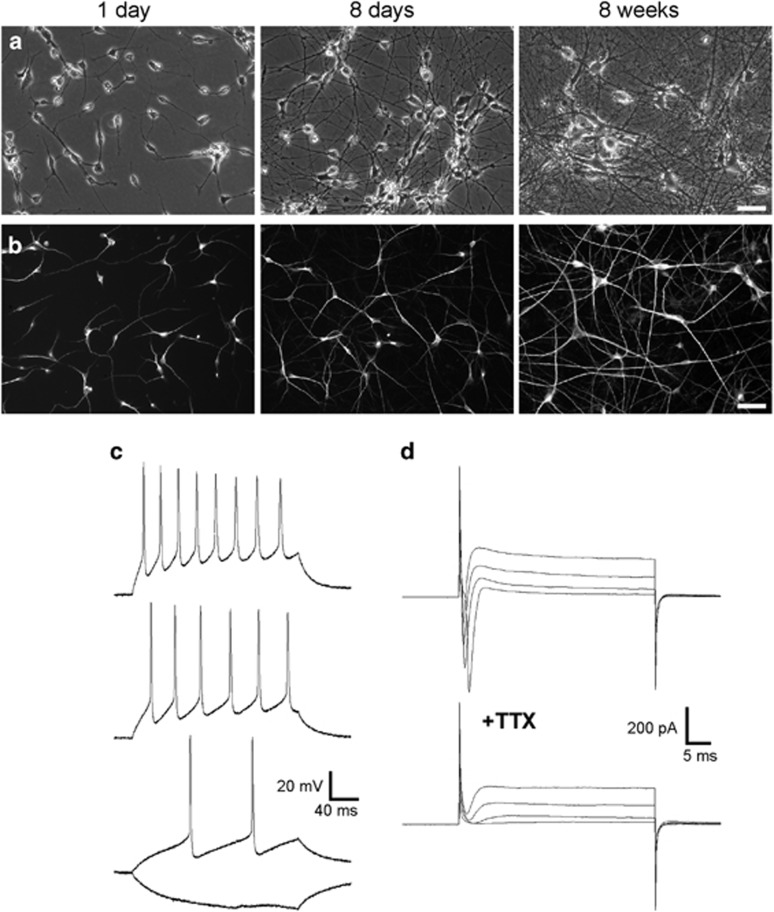
Electrical excitability of immunopurified human iPSC-derived neurons after *in vitro* maturation. (**a** and **b**) Photomicrographs (**a**) and MAP2 immunostainings (**b**) of immunopurified iPSC-derived neurons show progressive morphological/dendritic maturation of neurons at different culture stages (time after immunopanning indicated). Note the extensive formation of axonal and dendritic processes. Scale bars=50 *μ*m. (**c**) Typical action potential discharges upon depolarizing current injections (40, 80, 120 pA; 8 weeks after immunopanning). (**d**) Voltage-dependent sodium and potassium ion currents elicited by step depolarizations (to −40, −20, 0, +10 mV; top). Holding potential: −60 mV. Addition of TTX (1 *μ*M) blocks selectively fast inward Na^+^ currents (bottom)

Further cultivation of the MAP2-positive immature human neurons led to the formation of extended neurites after 1 week and to the formation of a dense neuritic network of mature neurons exhibiting enlarged somata at 8 weeks after immunopurification (Figure 2a). These cultures contained only very few glial cells due to the inhibition of proliferation of non-neuronal cells (see Materials and Methods). To study whether morphologically maturated iPSC-derived human neurons exhibit essential functional properties typical of cultured cortical neurons, we performed a basic electrophysiological characterization. Whole-cell patch-clamp recordings (at 8 weeks after immunopurification) of the membrane potential (57.8±0.7 mV resting potential) in current-clamp mode revealed the generation of action potentials upon injection of depolarizing current in all neurons tested (Figure 2b). To demonstrate the expression of voltage-dependent Na^+^ currents, we did whole-cell recordings in voltage-clamp mode at a holding potential of −60 mV. Step depolarizations of the membrane potential elicited typical inward Na^+^ currents that were blocked by addition of tetrodotoxin (TTX; 1 *μ*M; Figure 2c). Thus, electrophysiological analysis revealed essential functional properties such as electrical excitability and TTX-sensitive voltage-dependent Na^+^ currents in hiPSC-derived neurons at 8 weeks after immunopurification.

### Characterization of functional synapses in mature hiPSC-derived neurons purified by immunopanning

**Figure 3:**
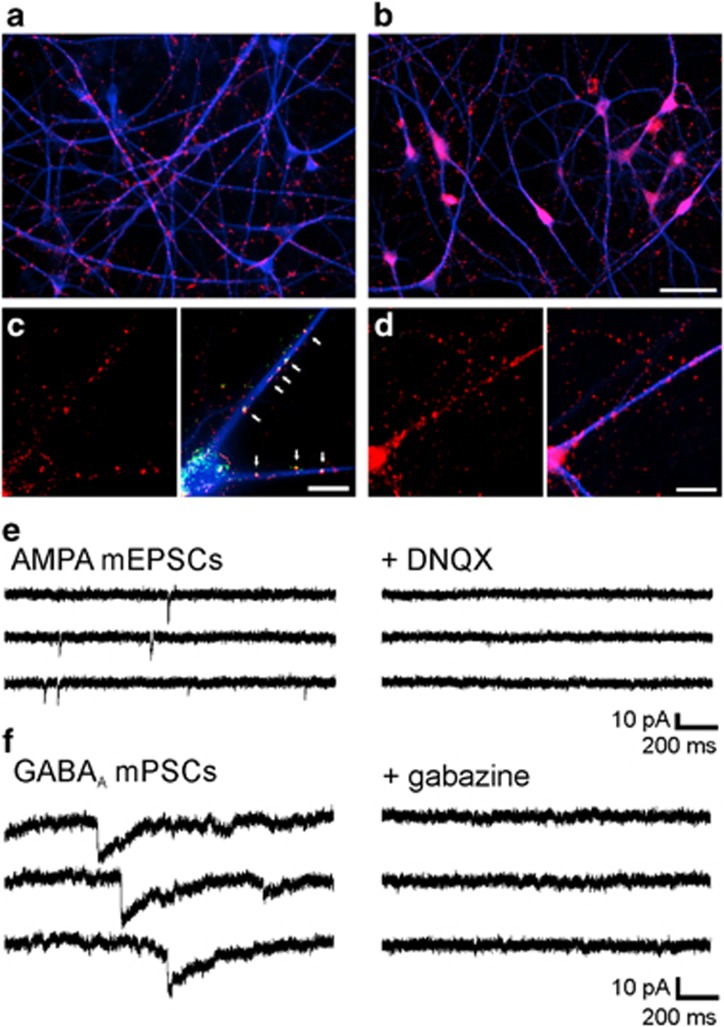
Formation of functional glutamatergic and GABAergic synapses in human iPSC-derived neurons at 8 weeks after immunopanning. (**a** and **b**) Synapsin I (**a**) and VAMP2 (**b**) immunostainings visualizing synaptic vesicle clusters (red). Dendrites were co-immunostained with MAP2 antibodies (blue). Note axonal vesicle clusters not contacting postsynaptic dendrites. Scale bar=50 *μ*m. (**c**) Punctate vGlut1 immunostaining (left; red) indicating the formation of glutamatergic synapses. vGlut1 immuno-positive vesicle clusters (red) on dendrites (right; MAP2 co-immunostaining, blue) co-localized with co-immunostained postsynaptic PSD-95 puncta (green, white arrows). Scale bar=20 *μ*m. (**d**) Punctate vGAT immunostaining (left; red) on postsynaptic dendrites (right; MAP2 co-immunostaining, blue) indicating the formation of GABAergic synapses. Scale bar=20 *μ*m. (**e**) Pharmacologically isolated (10 *μ*M gabazine, 1 *μ*M TTX) spontaneous AMPA mEPSCs recorded at a holding potential of −60 mV (left traces) were completely blocked by addition of DNQX (10 *μ*M; right traces) demonstrating the formation of functional glutamatergic synapses. (**f**) Pharmacologically isolated (10 *μ*M DNQX, 1 *μ*M TTX) spontaneous GABA_A_ mPSCs recorded at a holding potential of −60 mV (left traces) were completely blocked by addition of gabazine (10 *μ*M; right traces) demonstrating the formation of functional GABAergic synapses

At this stage of maturation, the formation of synaptic structures was indicated by vesicle clusters immunocytochemically stained for the synaptic vesicle-associated proteins VAMP2 (vesicle-associated membrane protein 2)/synaptobrevin and synapsin I on dendrites (Figures 3a and b). The presence of both glutamate and γ-aminobutyric acid (GABA) containing presynaptic vesicle clusters was confirmed by a punctate immunostaining on dendrites for the vesicular glutamate transporter 1 (vGlut1, colocalized with postsynaptic postsynaptic density protein 95 (PSD95) puncta) and for the vesicular GABA transporter (vGAT), respectively (Figures 3c and d). Moreover, the formation of functional synapses was indicated by spontaneous miniature postsynaptic currents (mPSCs) that were observed by whole-cell patch-clamp recording at −60 mV holding potential (Figures 3e and f). We further characterized spontaneous mPSCs pharmacologically by using specific antagonists. AMPA receptor-mediated miniature excitatory postsynaptic currents (mEPSCs) were isolated by addition of gabazine (10 *μ*M) and TTX (1 *μ*M), and were blocked by the AMPA receptor antagonist 6,7-dinitroquinoxaline-2,3-dione (DNQX; 10 *μ*M; Figure 3e). GABA_A_ receptor-mediated mPSCs were isolated by addition of DNQX and TTX and were blocked by addition of gabazine (Figure 3f). This demonstrates the presence of both functional glutamatergic and GABAergic synapses. Taken together, our findings demonstrate that at 8 weeks after immunopurification our iPSC-derived human neurons exhibit essential functional properties, such as excitability, synaptic activity, and functional glutamatergic and GABAergic synapses.

### A*β* induced loss of cycling vesicle clusters in iPSC-derived human neurons

To investigate whether functional synapses in iPSC-derived human neurons are susceptible to the deleterious effects of A*β*, we added A*β* contained in the supernatant from cultures of 7PA2 Chinese hamster ovary (CHO) cells (expressing human APP751 carrying the familial amyloid precursor protein (APP) V717F mutation)^5, 35, 36^ to hiPSC-derived neurons at 8 weeks after immunopurification. Conditioned medium from 7PA2 cells was diluted 1:1 with standard culture medium.^37^ To test for potential unspecific effects of the 7PA2 supernatant, we immunodepleted A*β* from the 7PA2 conditioned medium by using the anti-A*β* monoclonal antibody IC16.^37, 38^ A*β*-induced defects were analysed 8 days after A*β* application.

**Figure 4:**
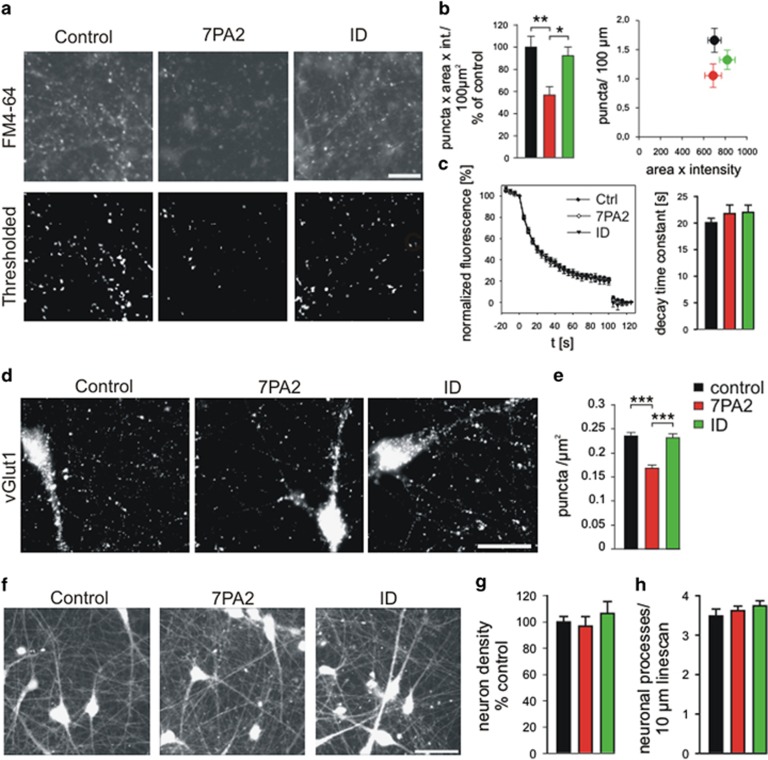
Incubation with A*β* impairs vesicle clusters in human iPSC-derived cortical neurons. (**a** and **b**) Cycling vesicle clusters in both axons and axon terminals were stained by the activity-induced uptake of the dye FM4–64. (**a**) Original fluorescence images (upper panel) and thresholded FM4–64-stained puncta (vesicle clusters; lower panel). Eight days incubation with A*β* containing 7PA2 supernatant (7PA2) or with immunodepleted 7PA2 supernatant (ID) or with vehicle (control). Scale bar=20 *μ*m. (**b**) Quantification of FM4–64 puncta fluorescence per image. Left: Total FM4–64 signal (number of puncta × mean total fluorescence intensity/punctum) per image. *n*=10/10/9 cultures. Normalization to control mean value of each cell preparation. Right: Density of FM4–64 puncta *versus* mean total intensity (area of punctum × average fluorescence intensity). Note the reduction in vesicle cluster density upon incubation with A*β*. (**c**) Activity-induced destaining of FM4–64 puncta by exocytosis of vesicles. Left: Destaining kinetics. Electrical stimulation (1200 stimuli at 20 Hz for 60 s) started at *t*=0. A second stronger stimulation was given at the end of the experiment (*t*=100 s) to achieve complete destaining (background fluorescence). Right: Quantification of decay time constants (*τ*). *n*=10/10/9 cultures. (**d**) vGlut1 immunostaining of vesicle clusters in both axons and axon terminals. Eight days incubation with A*β* containing 7PA2 supernatant (7PA2) or with immunodepleted 7PA2 supernatant (ID) or with vehicle (control). Scale bar=20 *μ*m. (**e**) Quantification of the density of vGlut1 immunostained puncta per image. *n*=16/24/15. (**f**) Calcein fluorescence staining of cells at the end of FM4–64 staining/destaining experiments. Scale bar=50 *μ*m. (**g**) Quantification of cell density by counting cell somata per image. *n*=10/10/8 cultures. (**h**) Linescans across neuritic processes were used to determine the density of processes in each image. *n*=10/10/8 cultures. Means±S.E.M.; *P<0.05, **P<0.01, ***P<0.001, one-way analysis of variance with *posthoc* Holm–Sidak test

First, we studied the effects of A*β* on cycling synaptic vesicle clusters in the processes of iPSC-derived human neurons. Cycling vesicle clusters were stained by the fluorescent dye FM4-64 (*N*-(3-triethylammoniumpropyl)-4-(6-(4-(diethylamino) phenyl) hexatrienyl) pyridinium dibromide).^39^ Uptake of extracellularly added FM4–64 (10 *μ*M) in recycling vesicles was stimulated by triggering the exocytosis and endocytosis of synaptic vesicles with extracellular electrical stimulation. After removal of extracellular FM4–64 dye, FM4–64-stained vesicle clusters were imaged by fluorescence microscopy (Figure 4a). For each FM4–64 staining experiment, the number of fluorescent puncta (i.e., vesicle clusters) was determined after thresholding the FM4–64 fluorescence image (see Figure 4a) and was multiplied with the mean total fluorescence intensity per punctum, resulting in an overall FM4–64 fluorescence signal per area of neuropil. Strikingly, 8 days after addition of A*β*, we observed a strong, significant reduction in the overall FM4–64 fluorescence signal (Figures 4a and b) indicating a toxic effect of A*β* on recycling vesicle clusters. Addition of immunodepleted 7PA2 supernatant did not affect the overall FM4–64 fluorescence signal demonstrating a specific effect of A*β* contained in the 7PA2-conditioned medium. The A*β*-induced reduction of the overall FM4–64 fluorescence signal was largely due to a reduction in the number of FM4–64-stained puncta (vesicle clusters) per area of neuropil as indicated by plotting FM4–64 puncta density *versus* the mean total fluorescence intensity per punctum (Figure 4b). We also studied the destaining of FM4–64-stained vesicle clusters by triggering vesicle re-exocytosis with extracellular electrical stimulation (1200 stimuli at 20 Hz (for 60 s)); Figure 4c). The mean destaining kinetics of FM4–64 puncta were not significantly affected by addition of A*β*, thus further supporting that a reduction in the number of vesicle clusters was the major A*β*-induced alteration.

To further confirm an A*β*-induced impairment of vesicle clusters, we immunocytochemically stained iPSC-derived human neurons for the synaptic vesicle marker vGlut1 after incubation with A*β*. Again, we observed a significant reduction in the density of vGlut1 immunopositive puncta (vesicle clusters) in neurites of A*β*-treated neurons as compared with vehicle-treated control cultures (Figures 4d and e). Addition of immunodepleted 7PA2 supernatant did not affect the number of vGlut1 puncta, again demonstrating a specific effect of A*β* contained in the 7PA2-conditioned medium. To check for A*β*-induced changes in the density of processes and in cell density, we fluorescently stained iPSC-derived human neurons by addition of the membrane permeable dye calcein-AM directly following the FM4–64 staining/destaining experiments (Figure 4f). Eight days after addition of A*β*, the number of neuronal cell bodies and the density of processes (determined by counting processes crossing a randomly chosen line) were not significantly affected (Figures 4g and h). In summary, our results indicate that addition of A*β* results in a strong reduction in the density of vesicle clusters in the neurites of iPSC-derived human neurons.

### A*β* induced functional impairment of glutamatergic synapses in iPSC-derived human neurons

**Figure 5:**
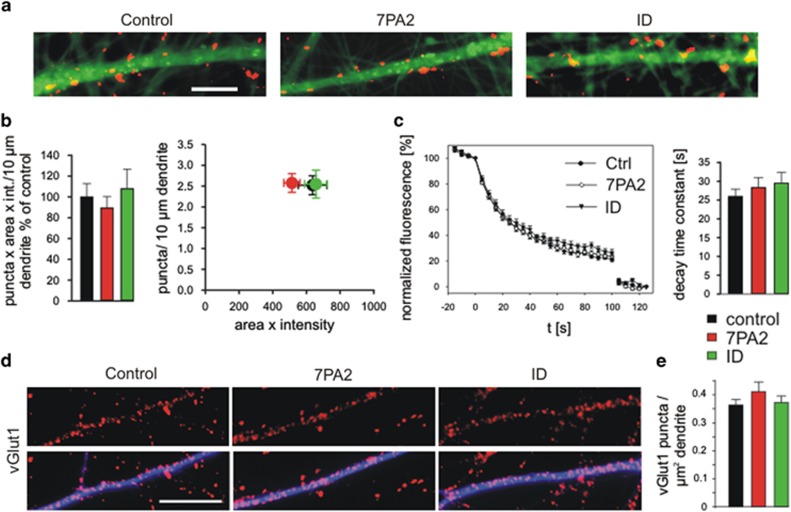
Presynaptic vesicle clusters are not affected by A*β* application. (**a**–**c**) Analysis of FM4–64-stained vesicle clusters on postsynaptic proximal dendrites (from experiments in Figure 4). (**a**) Thresholded FM4–64-stained puncta (vesicle clusters) on calcein-stained proximal dendrites are illustrated. Scale bar=10 *μ*m. (**b**) Quantification of FM4–64 puncta fluorescence on proximal dendrites. Left: Total FM4–64 signal (number of puncta × mean total fluorescence intensity/punctum) of puncta on a proximal dendrite. *n*=32/37/28 dendrites. Right: Density of FM4–64 puncta on a proximal dendrite *versus* mean total intensity (area of punctum × average fluorescence intensity). (**c**) Activity-induced destaining of FM4–64 puncta on proximal dendrites. Left: Destaining kinetics (for stimulation, see Figure 4). Right: Quantification of decay time constants (*τ*). *n*=32/37/32 dendrites. (**d** and **e**) Analysis of vGlut1 immunopositive vesicle clusters on MAP2 immunopositive postsynaptic dendrites (from experiments in Figures 4d and e). (**d**) vGlut1-positive puncta (vesicle clusters, upper panel) on dendrites immunostained for MAP2 are shown (lower panel). Scale bar: 10 *μ*m. (**e**) Quantification of the density of vGlut1 positive puncta on dendrites. *n*=24/23/24 dendrites. Eight days incubation with A*β* containing 7PA2 supernatant (7PA2) or with immunodepleted 7PA2 supernatant (ID) or with vehicle (control). Means±S.E.M.; one-way analysis of variance

We next studied whether the addition of A*β* induced deleterious changes at *bona fide* synaptic sites contacting postsynaptic dendrites. To focus on presynaptic vesicle clusters, we reanalysed the FM4–64 puncta (from the FM4–64 staining/destaining experiments described above) that contacted the proximal dendrites of iPSC-derived human neurons. Proximal dendrites arising from neuron somata were identified by calcein-AM staining of whole neurons after FM4–64 staining/destaining experiments, and FM4–64 puncta were analysed after thresholding the FM4–64 fluorescence image (Figure 5a). Eight days after addition of A*β*, we did not observe a significant change in the dendritic density of FM4–64 puncta or a significant change in total FM4–64 fluorescence intensity per punctum or a significant change in the overall presynaptic FM4–64 fluorescence signal per dendrite (Figure 5b). Furthermore, the quantitative analysis of the stimulation-induced destaining of FM4–64 puncta did not reveal any significant changes in exocytosis (Figure 5c). We also reanalysed the presynaptic vGlut1 immunopositive puncta (described above) located on dendrites, which were identified by MAP2 co-immunostaining of dendrites (Figure 5d). Again, 8 days after addition of A*β*, we observed no significant changes in the density of vGlut1 puncta on dendrites (Figure 5e). Taken together, the analysis of FM4–64 fluorescent and of vGlut1 immunopositive puncta on dendrites thus revealed no significant presynaptic alterations at a relatively early phase of incubation with A*β*, suggesting that *bona fide* presynaptic vesicle clusters and functional release properties were not affected by short-term application of A*β*.

**Figure 6:**
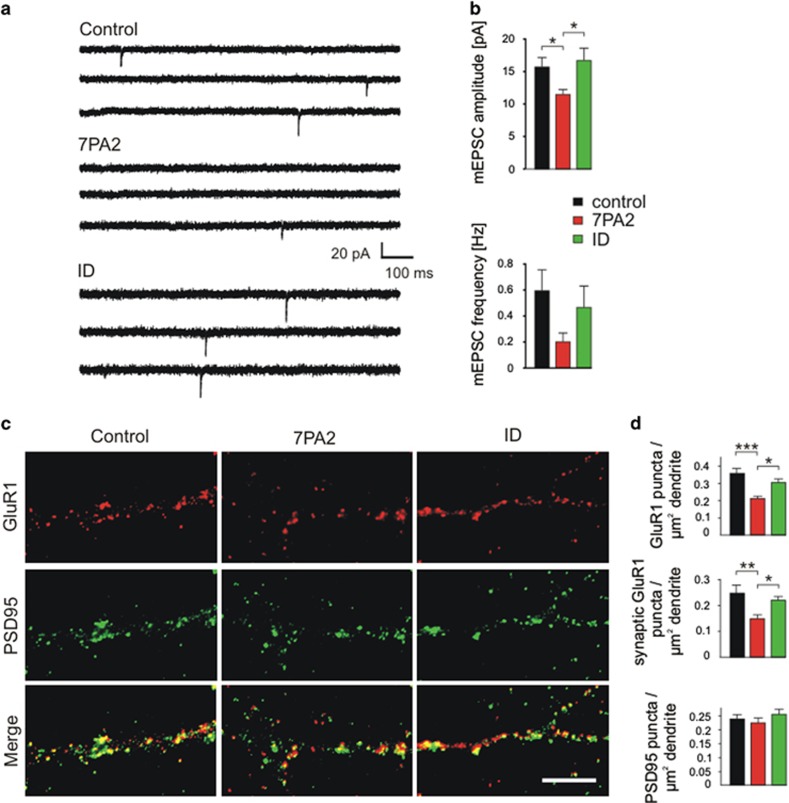
A*β* induced synapse damage in human iPSC-derived cortical neurons. (**a** and **b**) Examples of patch-clamp recordings of AMPA mEPSCs (**a**) and quantification of AMPA mEPSC amplitudes and frequencies (**b**). Eight days incubation with A*β* containing 7PA2 supernatant (7PA2) or with immunodepleted 7PA2 supernatant (ID) or with vehicle (control). *n* (cells) is 19/16/11 for mEPSC amplitudes and 26/28/19 for mEPSC frequency. Cells exhibiting no or <5 AMPA mEPSCs did not contribute to amplitude analysis. Note the A*β*-induced reduction in AMPA mEPSC amplitudes. (**c** and **d**) Immunocytochemical analysis of GluR1(GluA1) and PSD95 expression revealed A*β*-induced AMPA receptor impairment. (**c**) Immunocytochemical stainings for GluR1 (upper panel), PSD95 (middle panel), and merge of GluR1 and PSD95 stainings (lower panel). Punctate staining within a dendrite is shown. Scale bar=10 *μ*m. (**d**) Quantification of GluR1 immunopositive puncta per dendritic area (upper bar graph, *n* (dendrites) is 9/10/12), and quantification of synaptic (colocalized with PSD95) GluA1 puncta per dendritic area (middle bar graph, *n* (dendrites) is 9/10/12). Lower bar graph: quantification of PSD95 immunopositive puncta per dendritic area. *n* (dendrites) is 23/22/24). Eight days incubation with A*β* containing 7PA2 supernatant (7PA2) or with immunodepleted 7PA2 supernatant (ID) or with vehicle (control). Means±S.E.M.; *P<0.05, **P<0.01, ***P<0.001, one-way analysis of variance with *posthoc* Holm–Sidak test

We next addressed whether the deleterious effects of A*β* on functional properties of glutamatergic synapses, which are well described in rodent neurons, are also observed in iPSC-derived human neurons. We recorded AMPA receptor-mediated mEPSCs (AMPA mEPSCs) using the whole-cell patch-clamp technique (1 *μ*M TTX, 10 *μ*M gabazine added, holding potential: −60 mV). We again applied A*β* containing supernatant of the 7PA2 CHO cell line at 8 weeks after immunopurification and studied its effects on AMPA mEPSCs 8 days later. Addition of A*β* resulted in a strong, significant reduction of the mean amplitude of AMPA mEPSCs (Figures 6a and b), indicating a loss of postsynaptic AMPA receptor function. This amplitude reduction was not observed upon addition of 7PA2 supernatant immunodepleted of A*β*, demonstrating a specific A*β* action. Addition of A*β* did not lead to a significant reduction of the mean frequency of AMPA mEPSCs (Figures 6a and b). The observed trend to a reduced AMPA mEPSC frequency is likely to be caused by limitations in the detection of small amplitude minis. To further confirm an A*β* induced impairment of postsynaptic AMPA receptors, we performed immunocytochemical stainings for GluA1(GluR1) (AMPA receptor subunit 1), PSD95, and MAP2 (to visualize dendrites) in iPSC-derived human neurons. Eight days addition of A*β* resulted in a significant reduction of the density of GluA1 immunopositive puncta on MAP2-immunostained dendrites (Figures 6c and d). Furthermore, the density of synaptic GluA1 immunopositive puncta (colocalized with PSD95 puncta) was also significantly reduced 8 days after A*β* application (Figure 6d). The dendritic density of PSD95 immunopositive puncta was not significantly altered (Figures 6c and d). These A*β*-induced changes were not detectable upon addition of 7PA2 supernatant immunodepleted of A*β*, again demonstrating a specific A*β* action. Taken together, the observed A*β*-induced reduction of AMPA mEPSC amplitudes and of dendritic GluA1 expression indicates an early action of A*β* on postsynaptic AMPA receptors in iPSC-derived human neurons. Interestingly, as indicated by the lack of A*β* effects on vesicle clusters on dendrites, an early A*β*-induced presynaptic impairment was not detectable.

### A*β*-induced effects on cell survival, cellular stress and tau protein in hiPSC-derived neurons

**Figure 7:**
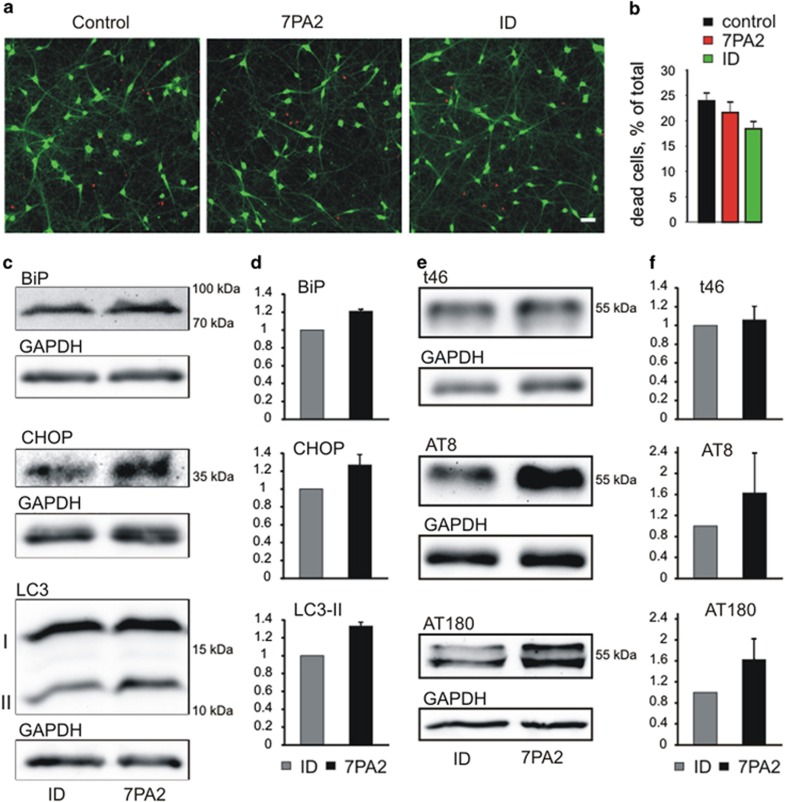
Effects of A*β* on cell survival, cellular stress, and tau protein. (**a**) Live/dead assay. Intact cells were stained with calcein (green) and nuclei without plasma membrane by Ethidium Homodimer 1 (red). (**b**) Quantification of dead cells. *n* (cultures) is 9/9/8. (**c** and **d**) Western blotting analysis of ER stress (BiP, CHOP) and autophagy (LC3-I and -II) markers. (**c**) Examples of western blots. 7PA2: cells incubated in A*β* containing 7PA2 supernatant for 8 days. ID: control cells incubated in immunodepleted 7PA2 supernatant. GAPDH: loading control. (**d**) Quantification (ratio to ID control (=1)) of A*β*-induced expression changes. *n*=3 independent A*β* applications. (**e** and **f**) Western blotting analysis of tau protein expression and phosphorylation. (**e**) Examples of western blots. t46, tau expression; AT8, AT180 phosphorylated tau. GAPDH: loading control. (**f**) Quantification (ratio to ID control (=1)) of A*β*-induced changes. *n*=3 independent A*β* applications. Note the A*β*-induced increase in tau phosphorylation. Means±S.E.M.

To study whether our short-term application of A*β* compromises cell survival, we stained healthy neurons and nuclei of disrupted cells, respectively by performing a live/dead assay (see Materials and Methods). Healthy neurons were fluorescently stained by addition of membrane permeable calcein-AM, which is intracellularly converted to fluorescent calcein. Nuclei without an intact plasma membrane were stained by Ethidium Homodimer 1 (Figure 7a). At 8 days of incubation, we did not observe significant effects of the addition of A*β* on cell survival (Figure 7b). Because A*β*-induced cellular stress might be detectable prior to cell death, we studied the expression of marker proteins for endoplasmic reticulum (ER) stress and autophagy by western blotting analysis. Interestingly, we found a slight A*β*-induced (8 days incubation) increase in the autophagy marker microtubule-associated protein 1A/1B-light chain II (LC3-II) and in the ER stress markers binding immunoglobulin protein (BiP) and CCAAT-enhancer-binding protein homologous protein (CHOP) (Figures 7c and d). This is in line with the recent description of increased ER stress in AD patient-derived iPSC lines upon neuronal differentiation.^40^ We further analysed A*β*-induced changes in tau protein expression and phosphorylation state by western blotting. Although the expression level of tau protein appeared not to be affected by short-term application of A*β* (8 days incubation), an increased phosphorylation of tau was clearly detectable (Figures 7e and f). This finding in human neurons is in line with the A*β*-induced phosphorylation of tau protein, which has been described in mouse models previously.^4, 41^

In summary, our results indicate that at an early phase of A*β* actions defects in synapse function are clearly revealed in iPSC-derived human neurons by changes in postsynaptic AMPA receptors. In addition, A*β*-induced alterations in neuritic vesicle clusters, in ER stress/autophagy marker proteins, and in tau protein phosphorylation were found. Our work thus establishes that iPSC-derived human neurons represent an innovative model system to study the molecular mechanisms of A*β*-induced synapse damage in AD.

## Discussion

In this study, we used hiPSCs to establish an innovative cell culture model of cortical neurons for specific aspects of AD. We analysed the effects of A*β* on human neurons, human synapses, and synaptic vesicle clusters. We observed a loss of axonal vesicle clusters, a loss of postsynaptic AMPA receptors, and increased tau protein phosphorylation, thus revealing complex deleterious actions of A*β*.

### *In vitro* differentiation of human iPSCs and immunopurification of iPSC-derived immature human neurons

*In vitro* differentiation of human pluripotent stem cells to neurons has been established by several groups.^22, 23, 42^ Without addition of specific morphogens/growth factors,^43^ iPSCs differentiate to dorsal, cortical-like neuronal progenitor cells characterized by Pax6 expression;^20, 22, 44^ however, other contaminating cell types are also generated. The invention of immunopanning protocols^34^ enables the highly efficient purification of specific types of neuronal precursors, neurons, or astrocytes.^45, 46, 47^ Similarly, the presence of NCAM has been used for selecting neuronally differentiated cells.^48, 49^ Here we have developed an immunopanning procedure based on NCAM antibodies, which yielded >90% MAP2-positive immature neurons. Immunocytochemical characterization of the purified neurons revealed typical cortical-like cultures consisting of mainly glutamaterigc and about 20% GABAergic neurons.

### Functional maturation of human iPSC-derived immature neurons, including synapse formation

In line with our results, it has been shown that neurons derived from human iPSCs generate action potentials and exhibit voltage-activated Na^+^ and K^+^ currents.^19, 24, 44, 50^ In addition, our iPSC-derived human neurons fired repetitive action potentials reminiscent of the firing pattern of regular spiking cortical pyramidal neurons.^24^ Similar to primary cultured rodent neurons,^51^ the formation of functional synapses has also been observed in human iPSC-derived neurons; however, reliable synapse formation and function appears to be difficult to achieve.^19, 25, 26^ Importantly, our iPSC *in vitro* differentiation protocol resulted reliably in the formation of both functional glutamatergic and GABAergic synapses, thus leading to synaptically active networks.

### Synaptotoxic effects of A*β* in human iPSC-derived neurons

We used our innovative model system for studying synaptic aspects of AD. Oligomeric A*β* peptides are thought to be crucial molecular entities in AD^1, 2, 3, 5, 52^ and exhibit—in rodent neurons—toxic effects at glutamatergic synapses leading ultimately to synapse loss.^8, 11, 37, 52, 53, 54, 55, 56^ The initial deleterious actions of A*β* on the function of glutamatergic synapses might be specifically localized either presynaptically or postsynaptically. In rodent neurons, an initial A*β*-induced removal of postsynaptic AMPA receptors by facilitating AMPA receptor endocytosis has been described.^9, 10, 12^ Furthermore, A*β* disrupts membrane trafficking and synaptic recruitment of AMPA receptors by reducing surface expression.^57^ In line with an initially postsynaptic mechanism, we found a pronounced A*β*-induced reduction in both the amplitudes of AMPAR-mediated mEPSCs and in postsynaptic AMPA receptor clusters in hiPSC-derived neurons. In addition, also presynaptic mechanisms of action of A*β* affecting both exocytosis and endocytosis have been proposed in rodent neurons.^10, 14^ However, our analysis of both FM-stained and immunocytochemically stained vesicle clusters apposed to dendrites in hiPSC-derived neurons did not reveal any short-term presynaptic effects of A*β*, thus indicating that A*β*-induced alterations in postsynaptic AMPA receptors might be of crucial importance in the human disease.

In addition to A*β*-induced synaptotoxicity, AD is characterized by the formation of neurofibrillary tangles and tau hyperphosphorylation.^58^ A*β*-induced tau phosphorylation^4, 13, 41^ has been proposed to be an early event leading to the formation of neurofibrillary tangles typical of tauopathy.^41^ In line with familial AD patient-derived iPSC-derived neurons,^33^ our data demonstrate that A*β*-induced tau phosphorylation occurs in hiPSC-derived neurons after short-term application of A*β*. Furthermore, we observed a slight A*β*-induced increase in proteins related to the ER stress-induced unfolded protein response (UPR). UPR includes enhanced expression of chaperones such as BiP, increased degradation of misfolded proteins, and activates autophagy.^59^ Failure of this system to restore ER homeostasis initiates apoptotic mechanisms. In line with our results, A*β* oligomers have been described to induce the UPR.^60^

### Effects of A*β* on vesicle clusters in hiPSC-derived neurons

Intriguingly, upon A*β* application we observed a reduced density of non-synaptic axonal vesicle clusters, which are well known to undergo exocytosis and endocytosis.^61, 62^ Because we did not observe any A*β*-induced changes in the FM destaining kinetics (reflecting exocytosis) and in the FM fluorescence intensity (reflecting endocytosis), an A*β*-induced impairment of the axonal transport of vesicles without axon degeneration appeared to underlie the reduction in the density of axonal vesicle clusters. In line with this idea, in rodent neurons an impairment of the axonal transport by A*β* has been observed.^15, 16, 17^ A*β*-induced defects in the transport of organelles, for example, mitochondria,^63^ and in the transport of dense core and synaptic vesicles^63, 64^ without axon degeneration have been described previously. Thus our results in iPSC-derived human neurons strongly support an important role of an A*β*-induced impairment in axonal transport of synaptic vesicles at early stages of the human disease.

### Conclusion: human iPSC derived neurons as an innovative model system to study toxic effects of A*β*

Here we have established hiPSC-derived neurons as an innovative AD model system to study the effects of A*β*. Toxic effects of A*β* were complex, including both non-synaptic effects on axonal vesicles and *bona fide* postsynaptic impairment of AMPA receptors. In addition, an A*β* induced increase in tau protein phosphorylation was found. Most importantly, our iPSC-derived human model system will aid the development of novel AD therapeutics based on the investigation of the molecular mechanisms of deleterious A*β* actions in human neurons.

## Materials and Methods

### hiPSC culture and neural differentiation

Experiments were approved by the local ethics committee of the University of Düsseldorf. Human iPSC line 8/25 (ref. 65) and in some experiments human iPSC line DF6–9–9T.B (WiCell, provided by Dr. J Thomson, University of Wisconsin^66^) were cultured according to standard procedures in mTeSr medium (STEMCELL Technologies, Köln, Germany) or in E8 medium^67^ on hESC qualified matrigel (Corning, Corning, NY, USA) coated cell culture six-well plates (Sarstedt, Nürnbrecht, Germany). Neural differentiation was performed according to a modified protocol of Li *et al.*^22^ EBs were allowed to form in T25 cell culture flasks (Sarstedt) in N2B27 medium containing 50% DMEM/F12 (Biochrom, Berlin, Germany), 50% Neurobasal, 2 mM Glutamax, 100 units/ml penicillin and 100 *μ*g/ml streptomycin, 0.1 mM *β*-mercaptoethanol, 1% B27 without vitamin A (all Gibco, Darmstadt, Germany), 3 *μ*g/ml heparin (Sigma Aldrich, St. Louis, MO, USA), and a modified N2 supplement with final concentrations of 7.5 *μ*g/ml human insulin, 50 *μ*g/ml BSA, 8 *μ*g/ml putrescine, 3.1 ng/ml progesterone, 2.6 ng/ml sodium selenite (all Sigma Aldrich), and 25 *μ*g/ml human holo-transferrin (Calbiochem, Darmstadt, Germany). After 14 days of suspension culture, EBs were plated on 100-mm cell culture dishes coated with growth factor reduced matrigel (Corning) in N2B27 medium. Six-to-8 weeks after starting differentiation, heterogeneous cultures were harvested for immunopanning by scraping in DPBS without calcium and magnesium (PanBiotech, Aidenbach, Germany).

### Immunopanning of hiPSC-derived immature neurons and neuronal maturation

Panning plates (100 mm, bacterial petri dish quality; Falcon/Corning, Darmstadt, Germany) were prepared by incubating goat anti mouse IgG+IgM antibody (Jackson Immunoresearch, Suffolk, UK) at 10 *μ*g/ml in 9 ml of 50 mM Tris HCl/pH 9.5 overnight at 4 °C. After three washing steps with DPBS, the panning plates were incubated overnight at 4 °C with the primary mouse anti-NCAM antibody (VIN-IS-53, kindly provided by Dr. Peter Andrews, University of Sheffield, UK) (0.55 *μ*g/cm^2^ in 0.2% BSA Fraction V (Gibco)/DPBS, 7 ml per plate). Plates were washed twice with DPBS and once with 0.02% BSA. Cells harvested from one 100-mm differentiation plate were enzymatically digested in accutase (Sigma Aldrich) for 30 min. After enzyme removal, cells were dissociated in 0.2% BSA in DPBS supplemented with 1 mg/ml glucose (Sigma Aldrich) and 0.33 mM sodium pyruvate (Gibco), pelleted at 200 g, resuspended in 0.02% BSA plus glucose and sodium pyruvate, and distributed to four panning plates (7 ml per plate). Cells were incubated on the panning plates for 10 min at RT. Non-adherent cells were removed by washing 10 times with DPBS. Adherent cells were detached by repeated pipetting (P1000, Eppendorf, Wesseling-Berzdorf, Germany) and rinsing of the plates with 0.02% BSA. Cells were pelleted at 130 × *g* for 10 min. Twenty-five thousand cells were plated on PO (1 mg/ml in borate buffer, coating overnight at RT) and laminin (10 mg/ml, coating 3 h at 37 °C) coated coverslips (12 mm, Assistant, Sondheim/Rhön, Germany) in a 10-*μ*l drop of neuronal culture medium, that is, Neurobasal, 2 mM Glutamax, 1 mM Sodium pyruvate, 100 units/ml penicillin, and 100 *μ*g/ml streptomycin (all Gibco), 2% NS21 supplement,^68^ 25 *μ*g/ml human insulin, 100 *μ*g/ml BSA, 16 *μ*g/ml putrescine, 62 ng/ml progesterone, 40 ng/ml sodium selenite (all Sigma Aldrich), 50 *μ*g/ml human holo-transferrin (Calbiochem), and 30 ng/ml BDNF (Peprotech, Hamburg, Germany). Two millilitres of medium was added after neurons had attached (30 min at 37 °C). To inhibit proliferation of neural stem cells, cultures were treated with 10 *μ*M cytosine *β*-D-arabinofuranoside hydrochloride (Sigma Aldrich) for the first 5 days. After removal of the mitotic inhibitor, half of the medium was changed once per week over a period of 2–3 months. Evaporation was compensated for by the addition of aqua bidest (Sigma Aldrich).

### A*β* containing 7PA2-conditioned medium and A*β* immunodepletion

All procedures were carried out as described.^37^ In brief, conditioned neuronal culture medium containing cell-secreted A*β* was obtained from a CHO cell line (7PA2) that expresses mutant human APP751 (V717F).^35, 36^ As determined by ELISA (A*β*_40_), 7PA2-conditioned medium contained A*β* at a concentration of 25–50 ng/ml. A*β* containing 7PA2-conditioned medium was immunodepleted with anti-A*β* antibody (IC16)^38^ coupled to NHS-Sepharose (GE Healthcare, Chalfont St Giles, UK) by overnight incubation at 4 °C followed by centrifugation. Depletion of A*β* was confirmed by immunoblotting.

### Immunocytochemistry and immunoblotting

Immunocytochemistry was carried out according to standard protocols, and quantification of images was carried out as described previously.^37^ Antibodies used are listed in Supplementary Table S1. DAPI (10 *μ*g/ml) was used to stain nuclei. Immunoblotting was performed according to standard protocols as described previously.^69^ Briefly, cells were scraped in 2 × loading buffer (200 mM Tris/HCl pH 6.8, 5% SDS, 20% glycerol, 0.04% bromphenol blue, 200 mM DTT), boiled for 5 min and centrifuged at 16.000 × *g* for 3 min. Lysates of different samples were adjusted for GAPDH content after analyzing triplicates by SDS-PAGE and immunoblotting. Adjusted samples were separated by SDS-PAGE (12%, Tris-Glycine) and blotted on nitrocellulose membranes. Immunoblotting and protein detection was performed according to standard procedures. Membranes were blocked with 10% Roti-Block (Carl Roth, Karlsruhe, Germany) in Tris-buffered saline with 0.1% Tween 20. Proteins of interest were visualized using Western Bright Quantum ECL (Biozym, Hessisch Oldendorf, Germany) and the Chemi Doc XRS System (Biorad, Munich, Germany).

### Calcein staining, fluorescence imaging, and analysis of cell death

For staining of healthy cells, we incubated cells with the calcein-green component (1/2000) of the LIVE/DEAD Kit (Molecular Probes, Darmstadt, Germany) for 10 min at 37 °C followed by immunocytochemistry (or direct imaging), which was performed according to standard procedures.^37^ Fluorescence imaging was carried out on a Zeiss Axiovert 200 M inverted fluorescence microscope (Zeiss, Oberkochen, Germany) equipped with a 12-bit monochrome CoolSNAP ES2 CCD camera (Photometrics, Tucson, AZ, USA) using the MetaVue and Metamorph (analysis) software (Visitron Systems, Puchheim, Germany). A*β*-induced cell death was studied by using the LIVE/DEAD Kit (Molecular Probes) according to the manufacturer's instructions.

### Electrophysiology and data analysis

Whole-cell patch-clamp recordings were carried out using an EPC7 patch-clamp amplifier (HEKA, Ludwigshafen/Rhein, Germany) and pCLAMP software (Molecular Devices, Sunnyvale, CA, USA) as described.^37, 70^ Intracellular solution contained 110 mM KCl, 0.25 mM CaCl_2_, 10 mM EGTA, and 20 mM HEPES, pH 7.3. The extracellular solution contained 130 mM NaCl, 5 mM KCl, 2.5 mM CaCl_2_, 1 mM MgCl_2_, and 20 mM HEPES, pH 7.3. Action potentials were elicited by depolarizing current injection in current-clamp mode (membrane potential −50 to −60 mV). Voltage-dependent currents (elicited by step depolarizations) and mPSCs were recorded in voltage-clamp mode (−60 mV holding potential). Quantitative analysis of AMPA mEPSCs was carried out using the Mini Analysis software (Synaptosoft, Decatur, GA, USA).

### Fluorescence imaging of cycling vesicle clusters

Cycling vesicle clusters were fluorescently stained by FM4–64 uptake.^39^ FM4–64 (10 *μ*M) was added to the extracellular solution (containing in mM: 119 NaCl, 2,5 KCl, 2 CaCl_2_, 2 MgCl_2_, 25 HEPES, 30 glucose, pH=7.4), and extracellular stimulation (400 stimuli at 20 Hz) was used to elicit exocytic/endocytic cycling of vesicles and FM4–64 uptake. Sixty seconds after stimulation, extracellular FM4–64 was removed by washing with extracellular solution (0 mM CaCl_2_ with addition of DNQX (10 *μ*M) and D-AP5 (50 *μ*M); 2 min wash, then 2 min wash in the presence of ADVASEP-7 (1 mM), and then 10 min wash), and fluorescent FM4–64 puncta were imaged.

For studying exocytosis of FM4–64 stained vesicles, destaining of FM4–64 puncta was elicited by extracellular electrical stimulation (1200 stimuli at 20 Hz (for 60 s)). After 100 s of destaining, a second stronger stimulation (2000 stimuli at 20 Hz without imaging (for 100 s)) was carried out to reach background fluorescence levels. Quantitative image analysis was carried out by defining regions of interest (ROI) around FM4–64 puncta. Total fluorescence intensity was determined for each FM4–64 punctum using the Metamorph software (Molecular Devices). Total background intensity was determined after the second stimulation within the same ROI and was subtracted from the total fluorescence intensity within the ROI to obtain the total FM4–64 fluorescence signal per punctum. Decay time constants of FM4–64 destaining were determined by monoexponentially fitting (SigmaPlot11 software, Systat Software Inc, San Jose, CA, USA) the mean fluorescence decay after averaging the normalized (intensity at the start of stimulation was set to 100%) destaining curves of FM4–64 puncta obtained from a field of view (Figure 4) or a proximal dendrite (Figure 5).

### Statistics

All data are given as means±S.E.M. Results were statistically analysed using one-way ANOVA with Holm–Sidak *posthoc* test (SigmaPlot11 software).

## Abbreviations

A*β*: amyloid-*β*
AD: Alzheimer's disease
AMPA: *α*-amino-3-hydroxy-5-methyl-4-isoxazolepropionic acid
APP: amyloid precursor protein
BiP: binding immunoglobulin protein
CHO: Chinese hamster ovary
CHOP: CCAAT-enhancer-binding protein homologous protein
Ctip2: chicken ovalbumin upstream promoter transcription factor-interacting protein 2
DNQX: 6,7-dinitroquinoxaline-2,3-dione
EB: embryoid body
EPSC: excitatory postsynaptic current
ER: endoplasmic reticulum
FM4–64: *N*-(3-triethylammoniumpropyl)-4-(6-(4-(diethylamino) phenyl) hexatrienyl) pyridinium dibromide
GABA: γ-aminobutyric acid
GAD67: glutamic acid decarboxylase 67
iPSC: induced pluripotent stem cell
LC3: microtubule-associated protein 1A/1B-light chain 3
MAP2: microtubule-associated protein 2
mPSC: miniature postsynaptic current
NCAM: neural cell adhesion molecule
Pax6: paired box protein 6
PSD95: postsynaptic density protein 95
Satb2: special AT-rich sequence-binding protein 2
Tbr1: T-box, brain, 1
TTX: tetrodotoxin
UPR: unfolded protein response
VAMP2: vesicle-associated membrane protein 2
vGAT: vesicular GABA transporter
vGlut1: vesicular glutamate transporter 1
