# Supplementary material for: Alzheimer's disease-related amyloid-β induces synaptotoxicity in human iPS cell-derived neurons
Source: Cell Death Dis. 2015 Apr 2;6(4):e1709–. doi: 10.1038/cddis.2015.72 (PMC4650541; doi:10.1038/cddis.2015.72)
Supplement: Supplementary Table 1 [file cddis201572x1.doc]

| **anti-** | **host** | **catalog no** | **company** | **dilution** |
| --- | --- | --- | --- | --- |
| Aß (4G8) | mouse | SIG-39200 | Covance | 1/200 for IP |
| AT8 | mouse | MN1020 | Thermos Scientific | 1/500 |
| AT180 | mouse | MN1040 | Thermo Scientific | 1/200 |
| BiP/GRP 78 | goat | Sc-1051 | Santa Cruz Biotech. | 1/800 |
| CHOP/GADD 153 | mouse | sc-7351 | Santa Cruz Biotech. | 1/200 |
| CTIP2 | mouse | ab18496 | Abcam | 1/200 |
| GAD67 (1G10.2) | mouse | MAB5406 | Millipore | 1/500 |
| GAPDH | mouse | CB1001 | Calbiochem | 1/150000 |
| GluR1 | rabbit | AB1504 | Millipore | 1/2000 |
| LC3B | rabbit | 2775 | Cell Signaling | 1/1000 |
| MAP2 | chicken | ab5392 | Abcam | 1/2000 |
| MAP2 | chicken | ab92434 | Abcam | 1/1000 |
| MAP2 | mouse | M9942 | Sigma Aldrich | 1/2000 |
| Nanog | rabbit | 9659 | Cell Signalling | 1/400 |
| N-Cadherin (32) | mouse | 610920 | BD Trans. Lab. | 1/100 |
| NCAM | mouse | VIN-IS-53 | DSHB/Peter Andrews | 1/200 |
| OCT4 | rabbit | 9659 | Cell Signalling | 1/400 |
| PAX6 | mouse | PAX6 | DSHB | 1/400 |
| PSD95 (6G6-1C9) | mouse | ab2723 | Abcam | 1/400 |
| SATB2 | mouse | ab51502 | Abcam | 1/50 |
| SOX1 | rabbit | AB15766 | Millipore | 1/400 |
| SOX2 | rabbit | 9659 | Cell Signalling | 1/400 |
| SSEA4 | mouse | 9659 | Cell Signalling | 1/400 |
| Synapsin 1 | mouse | 106001 | Synaptic Systems | 1/2000 |
| Synaptobrevin 2 | mouse | 104 211 | Synaptic Systems | 1/500 |
| Synaptobrevin 2 | rabbit | 104 202 | Synaptic Systems | 1/2000 |
| TBR1 | rb IgG | ab31940 | abcam | 1/500 |
| Tau46 | mouse | 4019 | Cell Signaling | 1/2000 |
| TRA-1-60 | mouse | 9659 | Cell Signalling | 1/400 |
| vGAT | rabbit | 131002 | Synaptic Systems | 1/1000 |
| vGLUT 1 | rabbit | 135 303 | Synaptic Systems | 1/3000 |

| **reactivity** | **host** | **conjugate** | **catalog no** | **company** | **stock** |
| --- | --- | --- | --- | --- | --- |
| chicken | goat | AMCA | 103-155-155 | Jackson Immuno Research | 1/1000 |
| chicken | goat | FITC | 46969-500 | abcam | 1/1000 |
| mouse | goat | Alexa 488 hca | A11029 | Invitrogen | 1/1000 |
| mouse | goat | Alexa 555 hca | A21424 | Invitrogen | 1/1000 |
| mouse | goat | AMCA | 115-155-146 | Jackson Immuno Research | 1/1000 |
| rabbit | goat | Alexa 488 hca | A11034 | Invitrogen | 1/1000 |
| rabbit | goat | Alexa 555 hca | A21429 | Invitrogen | 1/1000 |
| mouse | goat | HRP | sc-2055 | Santa Cruz Biotech | 1/4000 |
| rabbit | goat | HRP | sc-2054 | Santa Cruz Biotech | 1/10000 |
| goat | horse | HRP | PI-9500 | Vector Laboratories | 1/4000 |

**Supplementary Table 1:** List of antibodies used.
