# Supplementary figures and images for: Alzheimer's disease-related amyloid-β induces synaptotoxicity in human iPS cell-derived neurons
Source: Cell Death Dis. 2015 Apr 2;6(4):e1709–. doi: 10.1038/cddis.2015.72 (PMC4650541; doi:10.1038/cddis.2015.72)

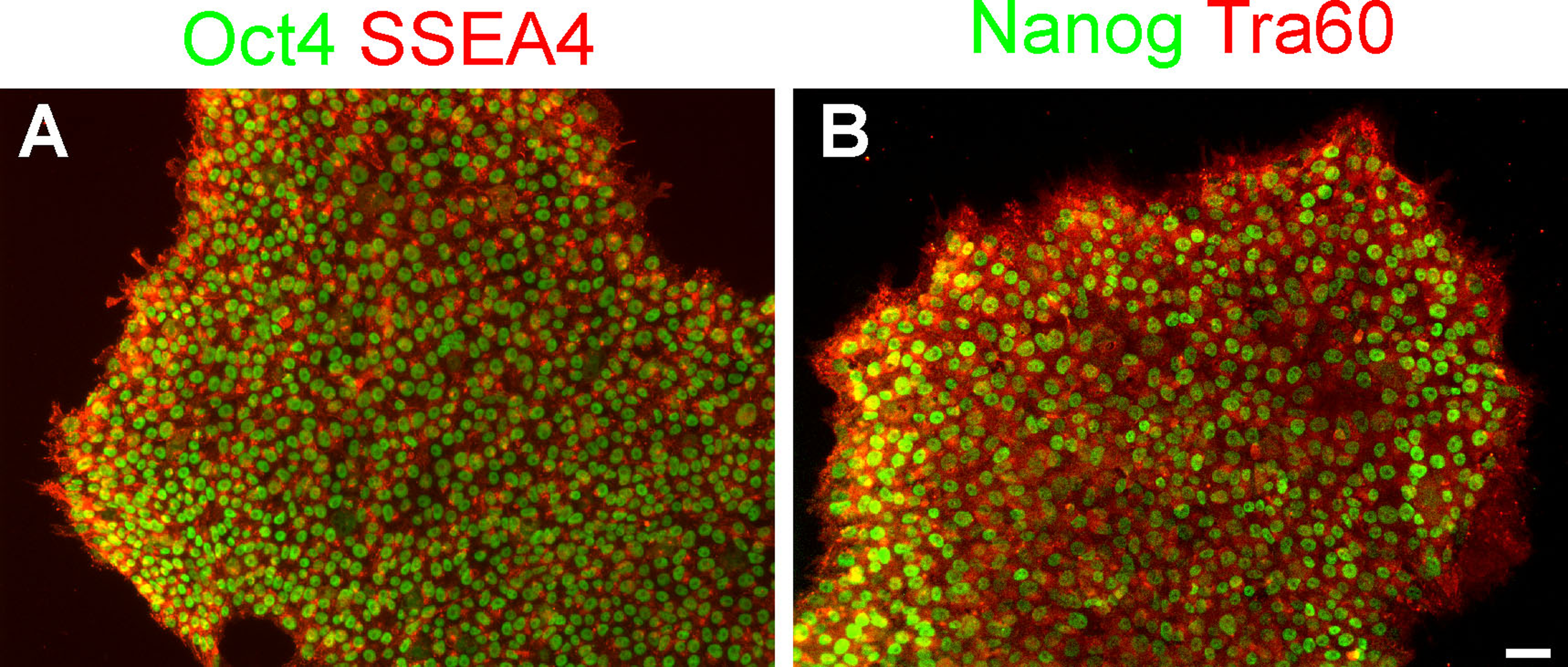

Supplement: Supplementary Figure 1 [file cddis201572x2.tif]

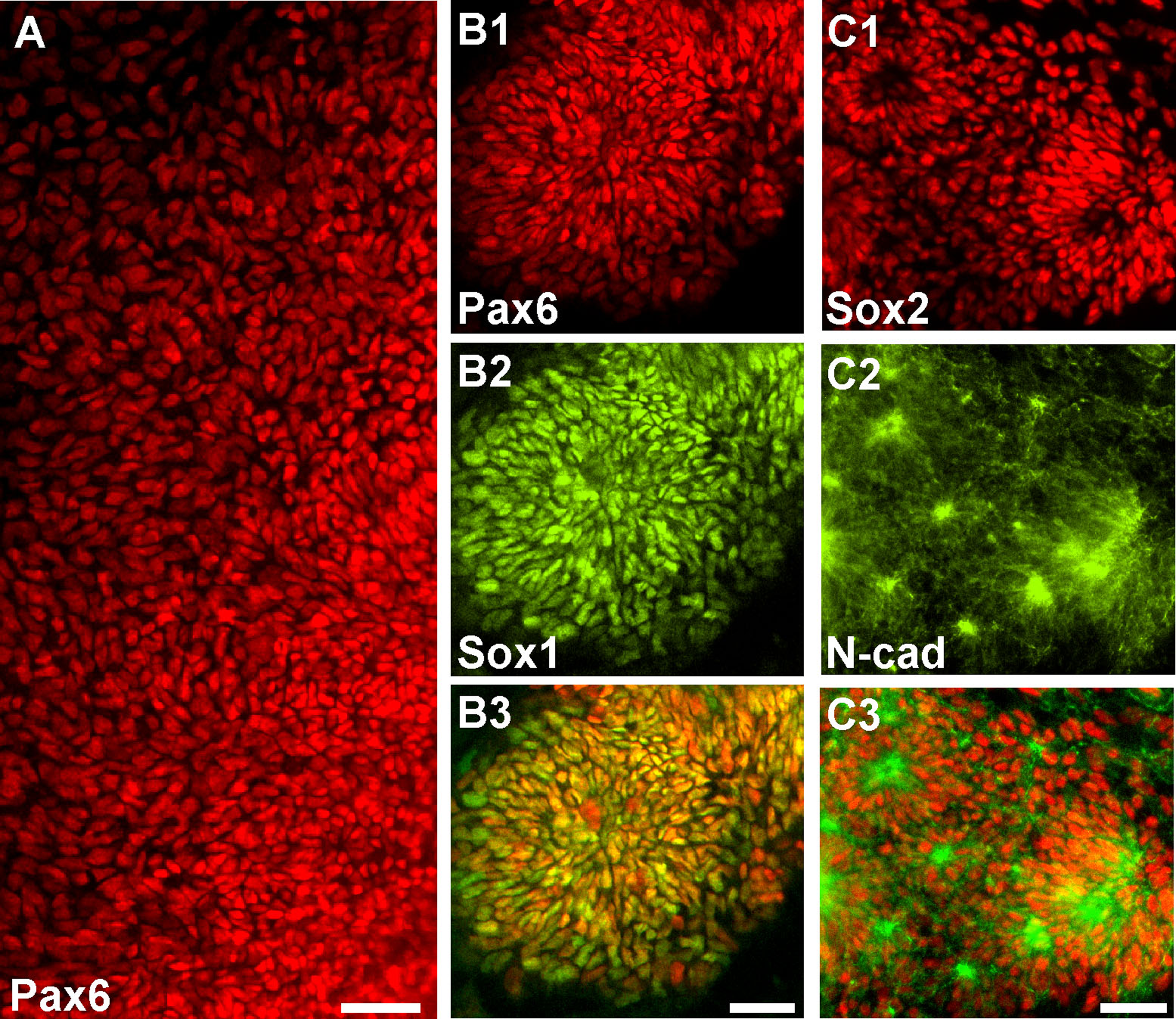

Supplement: Supplementary Figure 2 [file cddis201572x3.tif]
