# Supplementary material for: Alzheimer's disease-related amyloid-β induces synaptotoxicity in human iPS cell-derived neurons
Source: Cell Death Dis. 2015 Apr 2;6(4):e1709–. doi: 10.1038/cddis.2015.72 (PMC4650541; doi:10.1038/cddis.2015.72)
Supplement: Supplementary Figure Legends [file cddis201572x4.doc]

**Supplementary figure legends**

**Suppl. Figure 1** Immunocytochemical characterization of human induced pluripotent stem cells. (**A, B**) Human iPS cells (see Zaehres et al., 2010) were immunostained for pluripotency markers (**A**) Oct4 (green), SSEA4 (red), and (**B**) Nanog, Tra60. Scale bar = 50 µm.

**Suppl. Figure 2** Immunocytochemical characterization of human iPSC-derived neuroepithelial cells. (**A**) Pax6 immunostaining of neuroepithelial cells (23 days of in vitro differentiation). (**B, C**) Neuroepithelial rosettes immunostained for Pax6 (**B1**) and Sox1 (**B2**), and Sox2 (**C1**) and N-cadherin (**C2**). (**B3, C3**) Overlays. Scale bars = 50 µm.
